# Attention-deficit/hyperactivity disorder and behavioural planning deficiencies in South African primary school children

**DOI:** 10.4102/sajpsychiatry.v26i0.1411

**Published:** 2020-10-22

**Authors:** Tshikani T. Boshomane, Basil J. Pillay, Anneke Meyer

**Affiliations:** 1 Department of Behavioural Medicine, Faculty of Health Sciences, University of KwaZulu-Natal, Durban, South Africa

**Keywords:** attention-deficit/hyperactivity disorder, behavioural planning, developmental disorder, primary school children, hyperactive

## Abstract

**Background:**

Attention-deficit/hyperactivity disorder (ADHD) is defined as a cognitive or behavioural developmental disorder. Inattentiveness, overactivity and impulsivity are regarded as the main clinical symptoms of ADHD. These symptoms may occur together or separately resulting in three recognised presentations: predominantly inattentive, predominantly hyperactive–impulsive and combined presentations.

**Aim:**

This study investigated deficiencies in behavioural planning in South African primary school children with and without ADHD.

**Setting:**

Tzaneen area in Limpopo province, South Africa.

**Methods:**

A total of 156 children (78 with ADHD and 78 matched controls without ADHD) of both genders, who were medication naïve and aged 6–15 years, participated in the study. The performance of the two groups was compared on a test of planning and problem-solving, the Tower of London (ToL) task. The results were analysed as a function of gender, age and ADHD presentation.

**Results:**

Children with ADHD especially ADHD-PI and ADHD-C used significantly more moves and took a longer time to complete the task than the controls (*p* < 0.001). There were no significant differences in the number of moves and time taken by the predominantly hyperactive-impulsive presentations of ADHD when compared to the controls. Gender and age did not influence the performance.

**Conclusion:**

The results showed that children with ADHD showed significantly more deficits mainly the ADHD-PI and ADHD-C presentations, which indicates that inattention is mainly responsible for deficiencies in behaviour planning. The ADHD-HI presentations and the control group were not affected.

## Introduction

Attention-deficit/hyperactivity disorder (ADHD) is a common heterogeneous neurodevelopmental disorder of genetic origin, with a childhood onset and which often persists into adulthood.^1,2^ According to the Diagnostic and Statistical Manual of Mental Disorders, Fifth Edition (DSM-5), ADHD is characterised by symptoms of overactivity, impulsivity and inattention, which are presently regarded as the main clinical symptoms.^3^

Globally, the prevalence of ADHD does not significantly differ between America, Australia and Africa.^2,4^ The prevalence of ADHD in children and adolescents worldwide is estimated to be 5.3%.^2^ In South Africa, Meyer et al.^5^ found that the prevalence of ADHD among primary school children of all ethnic populations in the Limpopo province to be 5.5%.

In children and adolescents, ADHD predominantly affects males with a male–female sex ratio of 4:1 in clinical samples and 2.4:1 in population studies.^2^ Attention-deficit/hyperactivity disorder is found to be as prevalent and with similar same-sex ratios on the African continent as in Western countries.^2,5^

It is widely recognised that ADHD is not only a behaviour disorder characterised by hyperactivity, impulsivity and inattention, but it is fundamentally a cognitive disorder, involving a developmental impairment of executive functions (EFs), which are the controlling system of the brain.^6^ Executive functioning is a complex cognitive control process, which enables self-regulation and self-directed behaviour towards a goal; modifies behaviour in the light of new information; makes decisions and evaluates risks, plans for the future, prioritises and sequences actions, and solves novel problems.^7^

Symptoms of ADHD arise from a primary deficit in specific EF domains, such as response inhibition, working memory, set-shifting and planning.^1,2,6^ Children with ADHD show impairments in judgement, organisation, planning and decision-making as well as in behavioural disinhibition and cognitive flexibility.^8^ As a result, they experience problems with social skills, and exhibit low self-esteem, low frustration tolerance and impaired academic performance.^9^

Executive dysfunction is generally attributed to structural and functional frontal pathology.^1,10^ The ventromedial prefrontal cortex (PFC) is associated with complex decision-making and strategic planning, while dorsolateral (PFC) is linked to working memory and behaviour planning.^1,11,12^ There is abundant support for the role of the PFC in the ability to plan and carry out a strategy for completion. Neurotransmitter circuits are involved in EFs, with the dopamine system, especially, playing an essential part in planning and cognitive flexibility by supplying the PFC.^7,13,14^ Therefore, a hypofunctioning mesocortical dopamine branch will cause poor behaviour planning.^14^ In this study the focus was on behaviour planning. Children who are deficient in planning behaviour display insufficient problem-solving strategies, which, in turn, compromise their learning ability at school.

Planning can be described as the execution of goal-directed behaviour to predict and evaluate outcomes. Also identifying, organising steps and elements needed to carry out an intention.^15,16^ To plan, one must be able to conceptualise changes from present circumstances (look ahead, deal objectively with oneself concerning the environment and view the environment objectively). The planner must also be able to conceive of alternatives, weigh and make choices, and entertain both sequential and hierarchical ideas necessary for the development of a conceptual framework or structure that will give direction to the carrying out of a plan. Good impulse control and reasonably intact memory functions are also necessary in planning.^17^ Moreover, all of this conceptual activity requires a capacity for sustained attention.^18^ The literature suggests that children with ADHD often show poor performance on tasks that require strategic planning.^19^

A study by Kofman et al.^19^ stated that children with ADHD exhibited poor performance where planning or a proper strategy was needed. Pila-Nemutandani and Meyer.^20^, Schmitz et al.^21^ and Mokobane et al.^22^ noticed that children with the combined (ADHD-C) presentation were highly deficient in behavioural planning and faced more difficulties when compared to both the predominantly hyperactive-impulsive (ADHD-HI) and predominantly inattentive (ADHD-PI) presentations and neurotypically children. However, a study by Geurts et al.^23^ showed no deficiencies in children with ADHD and normal controls with regard to planning.

The purpose of the study was to investigate whether children with ADHD have deficits in behaviour planning as measured by the Tower of London (TOL)

## Research methods and design

### Study design

The study was conducted in primary schools in the Tzaneen area, Limpopo, during 2017. A quantitative, cross-sectional case–control study, experimental design was used. To establish whether children with ADHD are deficient in behaviour planning, the sample was divided into participants with ADHD and matched controls without symptoms of ADHD.

### Setting

The selected regions were in a rural area in which assessment of this nature is very rare. The areas were selected based on the remoteness of the regions so that the community could benefit from such research.

### Study population and sampling strategy

The sample (*n* = 156) was divided equally in terms of gender (76 boys and 80 girls), aged 6–15 years of age, were recruited from a school-based population in the Tzaneen area in the Limpopo province, South Africa. They were Sepedi and Xitsonga speaking, grade 1–grade 7 learners. The participants were divided into two age groups: 6–10 and 11–15 years. The sample was chosen because of its accessibility. To detect symptoms of ADHD, the learners were screened using the disruptive behavioural disorder (DBD) rating scale, based on ratings from parents and teachers.^24,25^ A total number of 5480 children were screened for ADHD using the DBD rating scale. Cut-off points were based on the results obtained from a previous study that had been conducted in Limpopo by Meyer et al.^5^ Learners who met the criteria for ADHD were assigned by the researcher as follows: participants with scores ≥ 17 on the hyperactivity and impulsiveness scale were classified as ADHD-HI presentations and those having a score ≥ 20 on the inattention scale were classified as ADHD-PI presentations, based on the epidemiological study by Meyer et al.^5^ Participants who met the criteria on both scales, ADHD-HI and ADHD-PI, were categorised as ADHD-C presentations. The cut-off point for the neurotypical control group was set at the 85th percentile or below to decrease the risk for false-positives in the group. Thus, children with scores of less than 15 on the hyperactivity and impulsivity scale and the inattention scale, matched for gender, age and home language, were selected as controls. Children were divided into an ADHD group and a control group, without ADHD symptoms. Seventy-eight children were found positive for ADHD symptoms (ADHD group) and they were matched for gender, age and home language with children without ADHD (control group, *n* = 78). Children with an intelligence quotient (IQ) lower than 80 and/or who are suffering from any head injury, epilepsy, cerebral palsy, cerebral malaria, autism spectrum disorder or severe psychiatric disorders, as reported on the demographic questionnaire by parents and confirmed by the teachers, were excluded from this study. At the time of testing, the researcher ensured that none of the children were taking psychostimulant medication.

### Data collection

Data were collected from primary school children within the Tzaneen area in Limpopo province during 2017. School principals gave their permission to conduct the study at their schools. Informed, written consent was obtained from the parents. The return rate of the questionnaires was 98%. The study was explained to all children selected for testing, and their assent was obtained. The assessments were conducted in the mornings. The children were assessed individually in a quiet room during school hours. The total number of correct responses for the TOL (total score) and the time taken to complete the specific response were recorded. The testing procedure for each child lasted 30 min and was conducted by a clinical psychologist and four research assistants (who held bachelor’s degrees in psychology) and were fluent in the child’s home language.

#### Instruments

**Screening instruments: Disruptive behaviour disorder rating scale.** The disruptive behaviour disorder rating scale (DBD), by Pelham et al.^24,25^ used to screen for ADHD symptoms, was completed by parents and teachers. The scale was standardised and normed for all language and population groups in Limpopo province, South Africa.^5^ The DBD assesses the presence and degree of ADHD-related symptoms (inattention and hyperactivity/impulsivity), oppositional defiant disorder (ODD) and conduct disorder (CD) as formulated in the DSM-IV-TR. The DBD has been translated into five South African languages, namely, Sepedi, Tshivenda, Afrikaans, Tsonga and Tswana, which are spoken in the Limpopo province. Internal consistency and norms for each language group have been established.^5^

Cronbach’s *α* for the primary school population in the Limpopo province was calculated at 0.90 for the HI scale and 0.92 for the inattention scale.^5^ For this study, the sample’s Cronbach’s *α* for the HI scale was calculated at 0.74, and at 0.79 for the inattention scale.

#### Assessment of behaviour planning

**The Tower of London:** The TOL is a widely used instrument for assessing planning ability. Shallice^26^ developed the instrument to assess higher order problem-solving capacity, specifically executive planning. It can also be used in a neuropsychological battery to assess motor planning and processing information.^27^ The TOL requires ‘forward thinking’ or planning and measures spatial planning and problem-solving because an early incorrect move can render the problem almost unsolvable.^28,29,30^ The TOL is considered to be a frontal lobe test which is believed to measure EFs involved with strategic planning.^31,32^ It has been proven to be sensitive in distinguishing planning behaviour deficits in children with ADHD and in neurotypically children.^20,33^

The apparatus consisting of three wooden beads (red, yellow and blue) had been placed. The researcher presented an example of the problems, on cards, which were to be solved in two to five steps. In a practice problem, the same initial position was used, where two steps were needed to reach a solution. The task consisted of 12 problems, which had been graded in terms of their difficulty. From the start position, the participants were expected to move the beads to the finish position shown on the cards, making use of the least number of steps. The reliability coefficients were split-half reliability, *r* = 0.72, and internal consistency, Cronbach’s *α* = 0.69.^17,34^

### Data analysis

Analysis of variance (ANOVA) was used to investigate possible between-group differences using the ToL raw scores. Analysis was done using Statistica 10 StatSoft.^35^ The results for each test were analysed with 4 × 2 (ADHD presentation × gender) ANOVA’s for independent samples. *Post hoc* tests consisted of multiple comparisons using the Bonferroni method.^36^

### Ethical consideration

We confirm that this manuscript is the original and has not been submitted to any other journal for publication. University of KwaZulu-Natal, is fully aware of this submission. Ethical approval to conduct the study was obtained (from the Biomedical Research Ethics Committee of the University of KwaZulu-Natal (Ethical Clearance Number HSS/1452/015D). In addition, permission was obtained from the Department of Education, Limpopo province, and school principals of the selected schools. Informed consent was obtained from the parents or legal guardians of the learners to participate in the study. The children also assented to their participation in the study. Participation in the study was voluntary, and participants were informed that they could withdraw at any stage.

## Results

The study hypothesised that children with ADHD will have impairments in behaviour planning when compared with neurotypically children. Age did not affect the results; therefore, it was not taken into account for further analysis. Statistically significant differences were found for the inattention scores (*p* < 0.001) and the HI scores (*p* < 0.001) of the DBD rating when the ADHD and control group were compared. The demographics and DBD scores for the sample are presented in Table 1.

**TABLE 1:** Demographics of the sample (*N* = 156).

Variables	ADHD				Control			
	*N*	%	*M*	SD	*N*	%	*M*	SD
**Gender**								
Male	38	24.4	-	-	38	24.4	-	-
Female	40	25.6	-	-	40	25.6	-	-
**Age group**								
6–10	15	9.6	9.47	1.19	15	9.6	9.60	1.18
11–15	63	40.4	12.24	1.25	63	40.4	12.32	1.36
**Language**				-				
Sepedi	72	46.2	-	-	72	46.2	-	-
Xitsonga	6	3.8	-	-	6	3.8	-	-
**Presentation**				-				-
ADHD-HI	12	7.7	-	-	-	-	-	-
ADHD-PI	30	19.2	-	-	-	-	-	-
ADHD-C	36	23.1	-	-	-	-	-	-
**Total**	**78**	**50**	**-**	**-**	**78**	**50**	**-**	**-**

ADHD, attention-deficit/hyperactivity disorder; ADHD-HI, hyperactive-impulsive presentation; ADHD-PI, predominantly inattentive presentation; ADHD-C, combined presentation; *M*, mean; SD, standard deviation.

The descriptive statistics, the results of the ANOVA and the *post hoc* analysis for the number of moves and the time taken in seconds on the TOL are shown in Table 2.

**TABLE 2:** Analysis of variance: Number of moves and time taken for the Tower of London.

Variables	ADHD *N* = 78 (50%)		ADHD-HI *N* = 12 (8%)		ADHD-PI *N* = 30 (19%)		ADHD-C *N* = 36 (23%)		Control *N* = 78 (50%)		Group comparison ANOVA DF (3, 148)			*Post hoc*	*p*
	*M*	SD	*M*	SD	*M*	SD	*M*	SD	*M*	SD	*F*	*p*	$\bar{x}$		
Moves	46.06	27.82	32.00	23.28	50.03	28.32	47.44	28.02	27.32	17.10	10.54	< 0.001	0.18	PI, C > Control	< 0.001
Time†	117.32	88.98	94.42	45.90	132.13	119.88	112.61	66.76	51.52	37.51	11.52	< 0.001	0.19	PI, C > Control	< 0.001

ADHD, attention-deficit/hyperactivity disorder (all presentations); ADHD-HI, hyperactive-impulsive presentation; ADHD-PI, predominantly inattentive presentation; ADHD-C, combined presentation; ANOVA, analysis of variance, *M*, mean; SD, standard deviation; DF, degree of freedom.

† , In seconds.

### Number of moves

There was neither main nor interacting effect for gender and age; therefore, the gender and age groups were analysed together. There was, however, a statistically significant difference in performance between the ADHD and control groups: *F*(3, 149) = 10.54, *p* < 0.001, $\bar{x}$ = 0.18. *Post hoc* analysis (Bonferroni) revealed that there was a statistically significant difference in performance between both the ADHD-PI and ADHD-C presentations and the control group (*p* < 0.001). The ADHD-PI and ADHD-C subtypes used significantly more moves than the controls. There was no significant difference in the number of moves used by the ADHD-HI subtype when compared to the controls.

### Time taken to complete the task

There was neither main nor interacting effect for gender and age. Therefore, the gender groups were not analysed separately. There was, however, a statistically significant difference in performance between the ADHD and control groups: *F*(3, 149) = 11.52, *p* < 0.001, $\bar{x}$ = 0.19. *Post hoc* analysis (Bonferroni) revealed that the differences between the ADHD-PI and ADHD-C and the control group were statistically significant, both at the *p* < 0.001 level. The children with ADHD-PI and ADHD-C took significantly more time than the controls to move the beads. There was no significant difference in the time taken by the ADHD-HI presentation when compared to the controls. Effect sizes ($\bar{x}$) of 0.18 (number of moves) and of 0.19 (time taken) are considered to be large.^37^

## Discussion

The study compared the performance of children with ADHD and a non-ADHD control group on measures of behaviour planning and problem-solving. No gender and age differences on task performance were found in this study. This was consistent with the study done by Biederman et al.^38^ and Seidman et al.^39^ who also found no significant differences between males and females with ADHD and neurotypically children. Several studies have reported that children with ADHD scored significantly lower on tests that measure especially planning, problem-solving, mental flexibility and spatial working memory than their matched peers.^8,40,41,42,43^ However, Houghton et al.^44^ did not find that the ToL discriminated between children with and without ADHD. This was also the conclusion of a Mexican study. Yanez-Tellez et al.^45^ found a great variety of cognitive deficiencies in children aged 7–12 years with ADHD, but the ToL could not differentiate between them and a control group without ADHD.

Chhabildas et al.^46^ have suggested that EF deficits in ADHD can be mainly accounted for by symptoms of inattention. Attention problems of ADHD usually occur in situations where stimuli are widely spaced in time. There are indications that development of functional units of behaviour, or performance of integrated behavioural sequences is hampered in children with ADHD when the task gets increasingly complicated or demands higher level processing.^47,48,49^ Children with ADHD has the inability to finish tasks, organise and sustain efforts as well as forgetfulness. The literature shows that they were found to be poor problem-solvers, by selecting the most relevant information included in the problems and they remembering smaller amounts of relevant and a greater amount of irrelevant information when compared to neurotypically children.^50,51^ This might be the result from changed motivational processes and they seemed to be evident when the ability to concentrate is stressed by the task being unwelcomed or uninteresting.^52^ According to Oosterlaan et al.,^53^ poor performance on the ToL is indicative of ADHD children making the first move before they had successfully generated an appropriate solution to the problem. Therefore, the fast planning times in ADHD children could be interpreted as impulsiveness which does not arise from a tendency towards fast motor response and equivalent to poor performance because of sustained inattention. The impulsive behaviour may aggravate the inattention problems that cause poor performance.

The results show that children with ADHD are significantly more impaired on measures of planning behaviour and problem-solving, especially the ADHD-PI and ADHD-C presentations when compared to ADHD-HI and the control group of non-ADHD children. The ADHD-C presentation’s poor performance indicated that children with symptoms of both inattention and hyperactivity-impulsivity are struggling to perform tasks by not being able to select strategies that entail reasoning. The findings were consistent with the study conducted by Sarkis et al.^54^ who found that the ADHD-C subtype performed significantly worse than the non-ADHD groups on the TOL. Similarly, Pila-Nemutandani and Meyer,^20^ Schmitz et al.,^21^ Mokobane et al.^22^ and Solanto et al.^55^ noticed that children with the ADHD-C presentations were deficient in behavioural planning and that they faced more difficulties when compared to both the ADHD-HI and ADHD-PI presentations and a control group of non-ADHD children. Saydam et al.^56^ also found that ADHD-C presentations had impaired planning strategies compared to the ADHD-PI presentation.

Generally, children diagnosed with ADHD-C struggle to remember instructions and to plan new strategies in a different situation.^56^ Children with ADHD-C had problems carrying out tasks to their conclusion and paying attention to instructions, and they were quick and disorderly when they plan their tasks.

However, the current study differs from the study by Houghton et al.^44^ who found that the TOL did not discriminate children with and without ADHD. On the contrary, Geurts et al.^23^ found conflicting results amongst children with ADHD-C and ADHD-PI; however, these children did not differ from the controls on any of the planning measures with increasing planning load. The incongruences between the Geurts et al.^23^ study and the findings of this study may be because of a smaller sample that they used. Further, the current research results differ with the findings of Barkley,^57^ who suggested that deficits in EF (behaviour planning and problem-solving) are related only to ADHD-C not to ADHD-PI and ADHD-HI. Barkley’s findings is supported by several other studies which show that ADHD-C is accompanied by more serious impairment than ADHD-PI.^58^ Therefore, the ADHD-C and the ADHD-PI presentations seemed to have more difficulty in planning ahead than the ADHD-HI and the non-ADHD comparison group.

The posterior parietal cortex is connected with the PFC and has been shown to represent neural correlates of decision-making and planning.^59^ These areas, especially the prefrontal areas, seem to be dysfunctional in children with ADHD, probably because of a hypofunctioning mesocortical dopamine branch, causing deficient attention and poor behavioural organisation.^14^

### Implication

Children with ADHD usually are hasty and disorderly when they plan behaviour.^58^ They have a compromised learning ability at school, which makes it difficult for them to apply new skills. It is essential that teachers and parents recognise children with ADHD early so that they can provide appropriate and effective intervention. Early referrals and necessary follow-up treatment at an early age is essential. In this regard, early pharmacological and/ or behavioural treatment should be provided when applicable.

### Limitations

The sample size was small, especially when the ADHD group was subdivided into three presentations. This may have influenced on the statistical outcome. Caution should be exercised when generalising these results to all South African children as the sample was homogeneous, consisting of rural Sepedi and Xitsonga speaking children only from the same geographical area. It is recommended for future studies to include more language groups. The TOL instructions should be standardised in different languages.

## Conclusion

The goal of the study was to assess the behaviour planning deficits in children with ADHD. The study showed that especially the inattentive and combined ADHD presentations have problems with planning, and problem-solving using the TOL.

## References

[1] Faraone SV Attention deficit hyperactivity disorder and premature death. Nat Rev Dis Primers. 2015;385(9983):2132–2133. 10.1016/S0140-6736(14)61822-5 25726517

[2] Polanczyk G, De Lima MS, Horta BL, Biederman J, Rohde LA The worldwide prevalence of ADHD: A systematic review and metaregression analysis. Am J Psychiatry. 2007;164(6):942–948. 10.1176/ajp.2007.164.6.942 17541055

[3] American Psychiatric Association Diagnostic and statistical manual of mental disorders: DSM-5. Washington, DC: American Psychiatric Association.

[4] Bakare M Attention deficit hyperactivity symptoms and disorder (ADHD) among African children: A review of epidemiology and co-morbidities. Afr J Psychiatry. 2012;15(5):358–361. 10.4314/ajpsy.v15i5.45 23044891

[5] Meyer A, Eilertsen D-E, Sundet JM, Tshifularo J, Sagvolden T Cross-cultural similarities in ADHD-like behaviour amongst South African primary school children. S Afr J Psychol. 2004;34(1):122–138. 10.1177/008124630403400108

[6] Ciuluvica C, Mitrofan N, Grilli A Aspects of emotion regulation difficulties and cognitive deficit in executive functions related of ADHD symptomatology in children. Procedia Soc Behav Sci. 2013;78:390–394. 10.1016/j.sbspro.2013.04.317

[7] Miyake A, Friedman NP The nature and organization of individual differences in executive functions: Four general conclusions. Curr Direct Psychol Sci. 2012;21(1):8–14. 10.1177/0963721411429458 PMC3388901 22773897

[8] Nigg JT, Blaskey LG, Huang-Pollock CL, Rappley MD Neuropsychological executive functions and DSM-IV ADHD subtypes. J Am Acad Child Adolesc Psychiatry. 2002;41(1):59–66. 10.1097/00004583-200201000-00012 11800208

[9] Rhodes MG, Kelley CM Executive processes, memory accuracy, and memory monitoring: An aging and individual difference analysis. J Mem Lang. 2005;52(4):578–594. 10.1016/j.jml.2005.01.014

[10] Willcutt EG, Doyle AE, Nigg JT, Faraone SV, Pennington BF Validity of the executive function theory of attention-deficit/hyperactivity disorder: A meta-analytic review. Biol Psychiatry. 2005;57(11):1336–1346. 10.1016/j.biopsych.2005.02.006 15950006

[11] Cortese S, Kelly C, Chabernaud C, Proal E, Di Martino A, Milham MP Toward systems neuroscience of ADHD: A meta-analysis of 55 fMRI studies. Am J Psychiatry. 2012;169(10):1038–1055. 10.1176/appi.ajp.2012.11101521 22983386 PMC3879048

[12] Gendle MH, Young EL, Romano AC Experimental. Effects of oral 5-hydroxytryptophan on a standardized planning task: Insight into possible dopamine/serotonin interactions in the forebrain. Clin Exp Hum. 2013;28(3):270–273. 10.1002/hup.2314 23609610

[13] Arnsten AF, Li B-M Neurobiology of executive functions: Catecholamine influences on prefrontal cortical functions. Biol Psychiatry. 2005;57(11):1377–1384. 10.1016/j.biopsych.2004.08.019 15950011

[14] Sagvolden T, Johansen EB, Aase H, Russell VA A dynamic developmental theory of attention-deficit/hyperactivity disorder (ADHD) predominantly hyperactive/impulsive and combined subtypes. Behav Brain Sci. 2005;28(3):397–418. 10.1017/S0140525X05000075 16209748

[15] Kaller CP, Unterrainer JM, Rahm B, Halsband U The impact of problem structure on planning: Insights from the Tower of London task. Cogn Brain Res. 2004;20(3):462–472. 10.1016/j.cogbrainres.2004.04.002 15268923

[16] Chiang H-L, Gau SS-F Impact of executive functions on school and peer functions in youths with ADHD. Res Dev Disabil. 2014;35(5):963–972. 24636025 10.1016/j.ridd.2014.02.010

[17] Kaller CP, Unterrainer JM, Stahl C Assessing planning ability with the Tower of London task: Psychometric properties of a structurally balanced problem set. Psychol Assess. 2012;24(1):46 10.1037/a0025174 21859218

[18] Lezak MD, Howieson DB, Loring DW, Fischer JS Neuropsychological assessment. 5th ed. New York, Oxford University Press; 2004.

[19] Kofman O, Gidley Larson J, Mostofsky SH A novel task for examining strategic planning: Evidence for impairment in children with ADHD. J Clin Exp Neuropsychol. 2008;30(3):261–271. 10.1080/13803390701380583 17852623

[20] Pila-Nemutandani RG, Meyer A Behaviour planning and problem solving deficiencies in children with symptoms of attention deficit hyperactivity disorder from the Balobedu culture, Limpopo province, South Africa. J Child Adolesc Mental Health. 2016;28(2):109–121. 10.2989/17280583.2016.1200582 27561999

[21] Schmitz M, Cadore L, Paczko M, Kipper L, Chaves M, Rohde LA Neuropsychological performance in DSM-IV ADHD subtypes: An exploratory study with untreated adolescents. Can J Psychiatry. 2002;47(9):863–869. 12500757 10.1177/070674370204700908

[22] Mokobane M, Pillay BJ, Meyer A Behaviour planning and inhibitory control in Sepedi-speaking primary school children with attention-deficit/hyperactivity disorder. S Afr J Psychol. 2020;50(1):11–23. 10.1177/0081246319838104

[23] Geurts HM, Verté S, Oosterlaan J, Roeyers H, Sergeant JA ADHD subtypes: Do they differ in their executive functioning profile? Archiv Clin Neuropsychol. 2005;20(4):457–477. 10.1016/j.acn.2004.11.001 15896560

[24] Pelham WE Jr, Gnagy EM, Greenslade KE, Milich RJ Teacher ratings of DSM-III-R symptoms for the disruptive behavior disorders. J Am Child Adolesc Psychiatry. 1992;31(2):210–218. 10.1097/00004583-199203000-00006 1564021

[25] Pillow DR, Pelham WE, Hoza B, Molina BS, Stultz CH Confirmatory factor analyses examining attention deficit hyperactivity disorder symptoms and other childhood disruptive behaviors. J Abnorm Child Psychol. 1998;26(4):293–309. 10.1023/A:1022658618368 9700521

[26] Shallice T, Biological Sciences Specific impairments of planning. Child Neuropsychol. 982;298(1089):199–209. 10.1098/rstb.1982.0082 6125971

[27] Foxcroft C, Roodt GJ An overview of assessment: Definition and scope. Oxford University Press, 2005; p. 3–7.

[28] Albert D, Steinberg LJ Age differences in strategic planning as indexed by the Tower of London. 2011;82(5):1501–1517. 10.1111/j.1467-8624.2011.01613 21679178

[29] Gau SSF, Shang CY Executive functions as endophenotypes in ADHD: Evidence from the Cambridge Neuropsychological Test Battery (CANTAB). J Am Acad Child Adolesc Psychiatry. 2010;51(7):838–849. 10.1111/j.1469-7610.2010.02215.x 20085608

[30] Unterrainer JM, Owen AM Planning and problem solving: From neuropsychology to functional neuroimaging. J Physiol Paris. 2006;99(4–6):308–317. 16750617 10.1016/j.jphysparis.2006.03.014

[31] Alvarez JA, Emory E Executive function and the frontal lobes: A meta-analytic review. Neuropsychol Rev. 2006;16(1):17–42. 10.1007/s11065-006-9002-x 16794878

[32] Beauchamp M, Dagher A, Aston J, Doyon J Dynamic functional changes associated with cognitive skill learning of an adapted version of the Tower of London task. Neuroimage. 2003;20(3):1649–1660. 10.1016/j.neuroimage.2003.07.003 14642475

[33] Scheres A, Oosterlaan J, Geurts H, Morein-Zamir S, Meiran N, Schut H Executive functioning in boys with ADHD: Primarily an inhibition deficit? Archiv Clin Neuropsychol. 2004;19(4):569–594. 10.1016/j.acn.2003.08.005 15163457

[34] Kaller CP, Debelak R, Köstering L, et al Assessing planning ability across the adult life span: Population-representative and age-adjusted reliability estimates for the Tower of London (TOL-F). Archiv Clin Neuropsychol. 2016;31(2):148–164. 10.1093/arclin/acv088 26715472

[35] StatSoft I STATISTICA (data analysis software system), version 10. 2011. Google Scholar; 2011.

[36] Weinfurt KP, Multivariate analysis of variance In Grimm LG. & Yarnold PR. editors, Reading and understanding multivariate statistics. Washington DC: American Psychological Association, 1995; p. 245–276.

[37] Cohen J Statistical power analysis for the behavioral sciences. 2nd ed. Hillsdale, NJ: Erlbaum; 1988.

[38] Biederman J Attention-deficit/hyperactivity disorder: A selective overview. Biol Psychiatry. 2005;57(11):1215–1220. 10.1016/j.biopsych.2004.10.020 15949990

[39] Seidman LJ, Valera EM, Makris NJ Structural brain imaging of attention-deficit/hyperactivity disorder. Biol Psychiatry. 2005;57(11):1263–1272. 15949998 10.1016/j.biopsych.2004.11.019

[40] Chiang H-L, Huang L-W, Gau SS-F, Shang C-Y Associations of symptoms and subtypes of attention-deficit hyperactivity disorder with visuospatial planning ability in youth. Res Dev Disabil. 2013;34(9):2986–2995. 10.1016/j.ridd.2013.06.020 23811280

[41] Culbertson WC, Zillmer EA The Tower of LondonDX: A standardized approach to assessing executive functioning in children. Archiv Clin Neuropsychol. 1998;13(3):285–301. 14590643

[42] Hughes C Finding your marbles: Does preschoolers’ strategic behavior predict later understanding of mind? Dev Psychol. 1998;34(6):1326. 10.1037//0012-1649.34.6.1326 9823515

[43] Tripp G, Ryan J, Peace K Neuropsychological functioning in children with DSM-IV combined type attention deficit hyperactivity disorder. Aust N Z J Psychiatry. 2002;36(6):771–779. 10.1046/j.1440-1614.2002.01093.x 12406119

[44] Houghton S, Douglas G, West J, Whiting K, Wall M, Langsford S Differential patterns of executive function in children with attention-deficit hyperactivity disorder according to gender and subtype. J Child Neurol. 1999;14(12):801–805. 10614567 10.1177/088307389901401206

[45] Yáñez-Téllez G, Romero-Romero H, Rivera-García L, Prieto-Corona B, Bernal-Hernández J, Marosi-Holczberger E Cognitive and executive functions in ADHD. Actas Esp Psiquiatr. 2012;40(6):293–298. 23165410

[46] Chhabildas N, Pennington BF, Willcutt EG A comparison of the neuropsychological profiles of the DSM-IV subtypes of ADHD. J Abnorm Child Psychol. 2001;29(6):529–540. 10.1023/A:1012281226028 11761286

[47] Sheppard D, Bradshaw JL, Georgiou N, Bradshaw JA, Lee PJ Movement sequencing in children with Tourette’s syndrome and attention deficit hyperactivity disorder. Mov Disord. 2000;15(6):1184–1193. 11104203 10.1002/1531-8257(200011)15:6<1184::aid-mds1018>3.0.co;2-n

[48] Kalff AC, De Sonneville LM, Hurks PP, et al Low and high-level controlled processing in executive motor control tasks in 5–6 year old children at risk of ADHD. J Child Psychol Psychiatry. 2003 10;44(7):1049–1057. 10.1111/1469-7610.00189 14531587

[49] Siklos S, Kerns KA Assessing multitasking in children with ADHD using a modified Six Elements Test. Archiv Clin Neuropsychol. 2004;19(3):347–361. 10.1016/S0887-6177(03)00071-4 15033221

[50] Pasolunghi MC, Cornoldi C, De Liberto SJ Working memory and intrusions of irrelevant information in a group of specific poor problem solvers. Mem Cognit. 1999;27(5):779–790. 10.3758/bf03198531 10540807

[51] Cornoldi C, Marzocchi GM, Belotti M, Caroli MG, Meo T, Braga CJ Working memory interference control deficit in children referred by teachers for ADHD symptoms. Clin Neuropsychol. 2001;7(4):230–240. 10.1076/chin.7.4.230.8735 16210212

[52] Taylor HE, Larson SJ Teaching children with ADHD–: What do elementary and middle school social studies teachers need to know? Soc Stud. 1998;89(4):161–164. 10.1080/00377999809599845

[53] Oosterlaan J, Scheres A, Sergeant JA Which executive functioning deficits are associated with AD/HD, ODD/CD and comorbid AD/HD+ ODD/CD? J Abnorm Child Psychol. 2005;33(1):69–85. 15759592 10.1007/s10802-005-0935-y

[54] Sarkis SM, Sarkis EH, Marshall D, Archer JJ Self-regulation and inhibition in comorbid ADHD children: An evaluation of executive functions. J Atten Disord. 2005;8(3):96–108. 10.1177/1087054705277265 16009658

[55] Solanto MV, Gilbert SN, Raj A, Zhu J, Pope-Boyd S, Stepak B Neurocognitive functioning in AD/HD, predominantly inattentive and combined subtypes. J Abnorm Child Psychol. 2007;35(5):729–744. 17629724 10.1007/s10802-007-9123-6 PMC2265203

[56] Saydam RB, Ayvaşik HB, Alyanak BJ Executive functioning in subtypes of attention deficit hyperactivity disorder. Archiv Neuropsychiatry. 2015;52(4):386 10.5152/npa.2015.8712 PMC5353113 28360745

[57] Barkley RA Behavioural inhibition, sustained attention, and executive functions: Constructing a unifying theory of ADHD. Psychol Bull. 1997;121(1):65 10.1037/0033-2909.121.1.65 9000892

[58] Schwenck C, Schmiedeler S, Zenglein Y, Renner T, Romanos M, Jans T Reflective and impulsive reactions in ADHD subtypes. Atten Defic Hyperact Disord. 2009;1(1):3–10. 10.1007/s12402-009-0002-6 21432574

[59] Faraone S, Asherson P, Banaschewski T, Biederman J, Buitelaar J, Ramos-Quiroga J Attention-deficit/hyperactivity disorder. Nat Rev Dis Primers. 2015;15020. 27189265 10.1038/nrdp.2015.20

